# The effect of transcutaneous electrical acupoint stimulation on pregnancy rates in women undergoing *in vitro* fertilization: a study protocol for a randomized controlled trial

**DOI:** 10.1186/1745-6215-15-162

**Published:** 2014-05-09

**Authors:** Cui Hong Zheng, Juan Zhang, Jing Wu, Ming Min Zhang

**Affiliations:** 1 Institute of Integrated Traditional Chinese and Western Medicine, Tongji Hospital, Tongji Medical College, Huazhong University of Science and Technology, 1095 Jiefang Avenue, Wuhan, Hubei 430030, China; 2 Reproductive Medical Center, Wuhan General Hospital of Guangzhou Military Command, 627 Wuluo Road, Wuhan, Hubei 430070, China; 3 Department of Epidemiology and Biostatistics, School of Public Health, Tongji Medical College, Huazhong University of Science and Technology, 13 Hangkong Road, Wuhan, Hubei 430030, China

**Keywords:** acupuncture, transcutaneous electrical acupoint stimulation, TEAS

## Abstract

**Background:**

The latest meta-analysis demonstrated that acupuncture improves pregnancy rates among women undergoing *in vitro* fertilization-embryo transfer (IVF-ET), and surface acupoint stimulation, such as transcutaneous electrical acupoint stimulation (TEAS), may have the same or better potential.

**Methods/Design:**

To explore the effect of TEAS on the clinical pregnancy rate (CPR) and live birth rate (LBR) compared with real acupuncture and controls in women undergoing IVF, a multicenter, randomized controlled trial will be conducted. The inclusion criteria are the following: infertile women <40 years of age undergoing a fresh IVF or intracytoplasmic sperm injection cycle, and the study will be restricted to women with the potential for a lower success rate as defined by two or more previous unsuccessful ETs (fresh or frozen). Those who have severe illnesses possibly precluding IVF or pregnancy, have FSH levels greater than 20 IU/L, received donor eggs, had been previously randomized for this study or had undergone acupuncture (in any modality) as infertility treatment will be excluded. The subjects will be randomly assigned to the TEAS group (IVF + TEAS), the electro-acupuncture (EA) group (IVF + EA), or the control group (only IVF). A total sample size of 2,220 women is required to detect differences in CPR among the three groups. TEAS or EA treatments will start once every two or three days from day 3 of menstruation in the ovarian stimulation cycle until the day of ET. The parameters of TEAS or EA will be the following: a frequency of 2/100 Hz, a moderate electrical current of 3 to 5 mA for TEAS and 0.8 to 1.0 mA for EA. The primary outcome is CPR. Secondary outcomes are LBR, the number of oocytes aspirated and the total gonadotropin dose used in the stimulation cycle.

**Discussion:**

This study will provide significant evidence for using a new method (TEAS) in IVF.

**Trial registration:**

ClinicalTrials.govID: NCT01608048 (05/24/2012).

## Background

*In vitro* fertilization-embryo transfer (IVF-ET) is the most successful infertility treatment, and for many people, this treatment represents the last possibility for pregnancy. However, the average IVF success rate per cycle using fresh, nondonor oocytes is still not satisfactory [1]. The majority of IVF cycles do not result in pregnancy. Although techniques such as controlled ovarian hyperstimulation (COH), *in vitro* fertilization, and embryo culture and transfer have greatly improved, certain patients are not successfully treated, even after several ETs.

Concurrently, the latent safety problems associated with using large doses of ovulation stimulants to obtain more eggs for IVF cannot be ignored [2]. Furthermore, IVF is an expensive procedure. Repeated cycles place a heavy financial burden on patients and their families. Therefore, it is important to maximize the efficiency of the procedure. Many patients have turned to complementary and alternative medical (CAM) treatments to increase the success rate of IVF. Among these CAM treatments, acupuncture is a frequently used adjunctive therapy.

Since the first report by Stener-Victorin *et al*. in 1999 [3] suggesting that acupuncture can increase the IVF clinical pregnancy rate (CPR), application of acupuncture to IVF has attracted considerable interest from the international community. In recent years, there have been a total of 23 randomized controlled trials (RCTs) evaluating acupuncture in IVF [3-25]. The latest comprehensive meta-analysis [26] of these trials demonstrated the following:

1. Acupuncture improved CPR among women undergoing IVF (Acu. versus Con.:39.5% versus 37.2%, OR = 1.21, 95% CI = (1.00, 1.46)).

2. The difference was more distinct when the types of controls and/or different acupuncture times were examined in a sensitivity analysis. Acupuncture at approximately the same time as COH is more suitable than when performed only concurrently with oocyte aspiration (OA) or embryo transfer (ET) (COH: Acu. versus Con., 31.5% versus 21.2%, OR = 1.75, 95% CI = (1.13, 2.71)).

3. Increasing stimulation intensity at the acupoint surface (as occurred in the Streitberger group), such as transcutaneous electro-stimulation, might induce the same or better therapeutic effects.

The results indicated that more positive effects from acupuncture during IVF can be expected if treatment times are appropriate, the treatment course is enough (at least four sessions), the means of intervention is improved, and syndrome differentiation and treatment according to individual characteristics is emphasized in future programs.

Transcutaneous electrical acupoint stimulation (TEAS) is a combination of transcutaneous electrical stimulation from the West and acupoints from traditional Chinese medicine, which is a method of inputting low frequency-pulse current into the body via the skin to treat diseases such as pain. Real acupuncture is often accompanied by some degree of discomfort or pain, and a portion of patients are afraid of acupuncture. However, this shortcoming of real acupuncture can be avoided by non-invasive stimulation (TEAS), and patients may find it more acceptable, both physically and mentally. Therefore, TEAS may have a more positive therapeutic effect. Zhang R demonstrated the positive effects of TEAS in women undergoing IVF [26], but there were no comparisons between TEAS and electro-acupuncture (EA).

Thus, the primary purpose of this study is to explore whether there are any differences between EA and TEAS in increasing pregnancy rates in women undergoing IVF compared with conventional IVF.

## Methods/Design

This study is designed as a multicenter, randomized, parallel-controlled, assessor- and statistician-blinded trial. Participants will be included from the following five hospitals: Affiliated Tongji Hospital, Union Hospital of Huazhong University of Science and Technology, Wuhan General Hospital of Guangzhou Military Command, Hubei Maternity and Child Care Hospital, and Renmin Hospital of Wuhan University. Each research center must be in strict accordance with the inclusion and exclusion criteria. The study will be conducted in the following sequence: patients will be enrolled after screening via the eligibility criteria, they will be randomized, they will undergo an approximate two-week treatment period, they will undergo a 40-week follow-up period, and assessment will follow. The research protocol has been approved by the Clinical Trial Ethics Committee of Tongji Medical College, Huazhong University of Science and Technology (approval number: (2012) (110)-1) and registered on the ClinicalTrials.gov Protocol Registration System (https://register.clinicaltrials.gov, ClinicalTrials.govID: NCT01608048). Written informed consent will be obtained from each participant. Patients will be recruited to join the study only once and will not receive any monetary compensation for study participation.

### Eligibility criteria

Women presenting to the reproductive medicine center of the involved hospitals will be screened for fitness to undergo IVF. All of the women with an indication for IVF, such as tubal infertility, severe male factor infertility, severe endometriosis, unexplained infertility, immune infertility, and anovulatory infertility, will be informed of the study.

The inclusion criteria are the following: (1) infertile women <40 years of age undergoing a fresh IVF or intracytoplasmic sperm injection (ICSI) cycle; (2) who have a potentially lower success rate, which is defined as two or more previous unsuccessful ETs (fresh or frozen); (3) who are willing to sign an informed consent form indicating that they are aware of the investigational nature of this study, which is in keeping with the institutional policies; and (4) who are willing to return to the study site for visits. The inclusion criteria were necessary because women who have experienced several unsuccessful OAs or ETs may easily accept randomization into this study, and adding TEAS or EA may significantly improve their clinical outcomes.

The exclusion criteria are the following: (1) have major medical illnesses (such as stage III heart disease, severe hypertension, uncontrolled diabetes mellitus, positive HIV status, severe bleeding dyscrasias, *etcetera*) possibly precluding IVF or pregnancy; (2) have FSH levels greater than 20 IU/L; (3) have received donor eggs; (4) have cutaneous lesions within the acupoint area; and (5) had previously participated in this study or previously undergone acupuncture (in whatever modality) as infertility treatment.

The withdrawal criteria are the following: (1) a patient is deemed unfit for the inclusion criteria; (2) early discontinuation for any reason (for example, consent withdrawal, adverse events, and treatment failure); (3) the patient does not strictly abide by the intervention protocol or uses other methods beyond the project that may affect pregnancy outcome; and (4) complete data missing for primary outcomes.

Patients have the right to decline participation in the study, and they may withdraw their consent at any time. Their consent or refusal to consent will not affect their expected IVF intervention.

### Sample size

Sample size calculation is based on the increased range of CPR from acupuncture as described in previous studies (13% to 18% based on the following studies: Stener-Victorin, 17.6% [3]; Paulus, 16.2% [4]; Westergaard, 16% [7]; Dieterle, 18% [8]; and Sator-Katzenschlager, 13.5% [25]). Smith CA chose a conservative 7% estimate based on their previous study [27]. The increased range of clinical effects from TEAS compared with the control group was 13.4% to 20.7% [28]. Therefore, we estimated a 7% increase for the EA group and a 15% increase for the TEAS group compared with the control group. The possible proportion of women with a clinical pregnancy for women with two or more cycle failures in the control group was 20% [27]. To detect differences using a pairwise comparison method, the sample size should be 1,850 according to optimum allocation (700 for the TEAS group, 700 for the EA group, and 450 for the control group) using the Shieh-O'Brien approximation method assuming that alpha (two-sided) is 0.05, the overall power is 0.90, the power between the TEAS and EA groups is 0.80, and the power between the EA and control group is also 0.80. A total sample size of 2,220 women (840 for the TEAS group, 840 for the EA group, and 540 for the control group) is required allowing for a 20% loss due to canceled cycles or no ET.

### Recruitment strategies and enrollment

A large number of infertile patients are currently seeking treatments at Chinese hospitals. Patients will be recruited mainly through advertisements placed via local media (television and newspaper) and recruitment posters in the reproductive center of the involved hospitals.

Interested patients will be screened by specific researchers who are very familiar with the study inclusion and exclusion criteria. The participants will be clearly and comprehensively told about the trial in which they will be participating such as the interests they will obtain, potential risks, trial group settings, and possible interventions they will receive. Baseline characteristics such as age, infertility type and duration, IVF indication and history, history of smoking or drinking, history of exposure to radiotherapy or chemotherapy, history of pelvic inflammatory disease (PID) or endometriosis, and history of previous pelvic surgeries will be noted. Patients who are willing to participate and meet the eligibility criteria will be introduced to the acupuncturist at the hospitals.

### Randomization and blinding

Included patients will be randomly divided into three groups: the TEAS group (conventional IVF + TEAS), the EA group (conventional IVF + EA), and the control group (only conventional IVF).

Fundamental characteristics of randomization include the inability of researchers to predict a patient’s assignment and no assignment changes after randomization. The randomization sequence will be generated by a computerized random number generator using the simple-randomization method (Excel, Microsoft Office 2007). A ‘patient’ database, which lists basic information such as patient name and contact details and a ‘randomization’ database, which contains data on patients who were registered in the trial, along with their allocations, will be generated. The ‘patients’ database is accessible to any researcher, whereas the ‘randomization’ database is password-protected; thus, it will be accessible only by the principal investigator and a nominated computer technician. Immediately before intervention, the acupuncturists will access the ‘patients’ database, type in the patient’s name and details, and hit an icon to randomize the patient. This act will lead to a dialog box asking the researcher to confirm that the patient is eligible. After the ‘okay’ button is pressed, patient information is automatically sent to the randomization database, where randomization takes place [29]. Grouping information will subsequently be sent to the acupuncturists for TEAS or EA treatments.

The assessor will assess the treatment outcome. The assessor will not be the acupuncture practitioner and will be blinded to the allocation results until the end of the study. The statistician will also be blinded to the concrete allocation until after the statistical analysis. The procedure will be performed with blinded staff as much as possible; however, the patients and the acupuncturists will not be blinded because the control group did not obtain sham TEAS or sham acupuncture.

### *In vitro* fertilization procedure

All included patients then will start IVF cycles using the long GnRH agonist protocol beginning on day 21 of the menstrual cycle until human chorionic gonadotropin (hCG) injection. Controlled ovarian stimulation will be performed from day 2 or 3 of the subsequent cycle with urinary or recombinant gonadotropin, the dose of which is individualized based on the anticipated response. The ovarian response will be monitored with ultrasound, and E2 levels will be estimated. The hCG trigger will be given on the day when three or more developing follicles 18 mm in size or larger are observed on transvaginal sonography. The total gonadotropin dose used to achieve the response as well as E2 levels on the day of hCG administration will be noted. Oocytes will be aspirated 36 h after hCG administration under transvaginal ultrasound guidance by experienced doctors. The ovarian response is defined as the number of oocytes obtained during the oocyte aspiration procedure. ET will be performed 72 h after oocyte aspiration.

### Acupuncture intervention(s)

**Figure 1:**
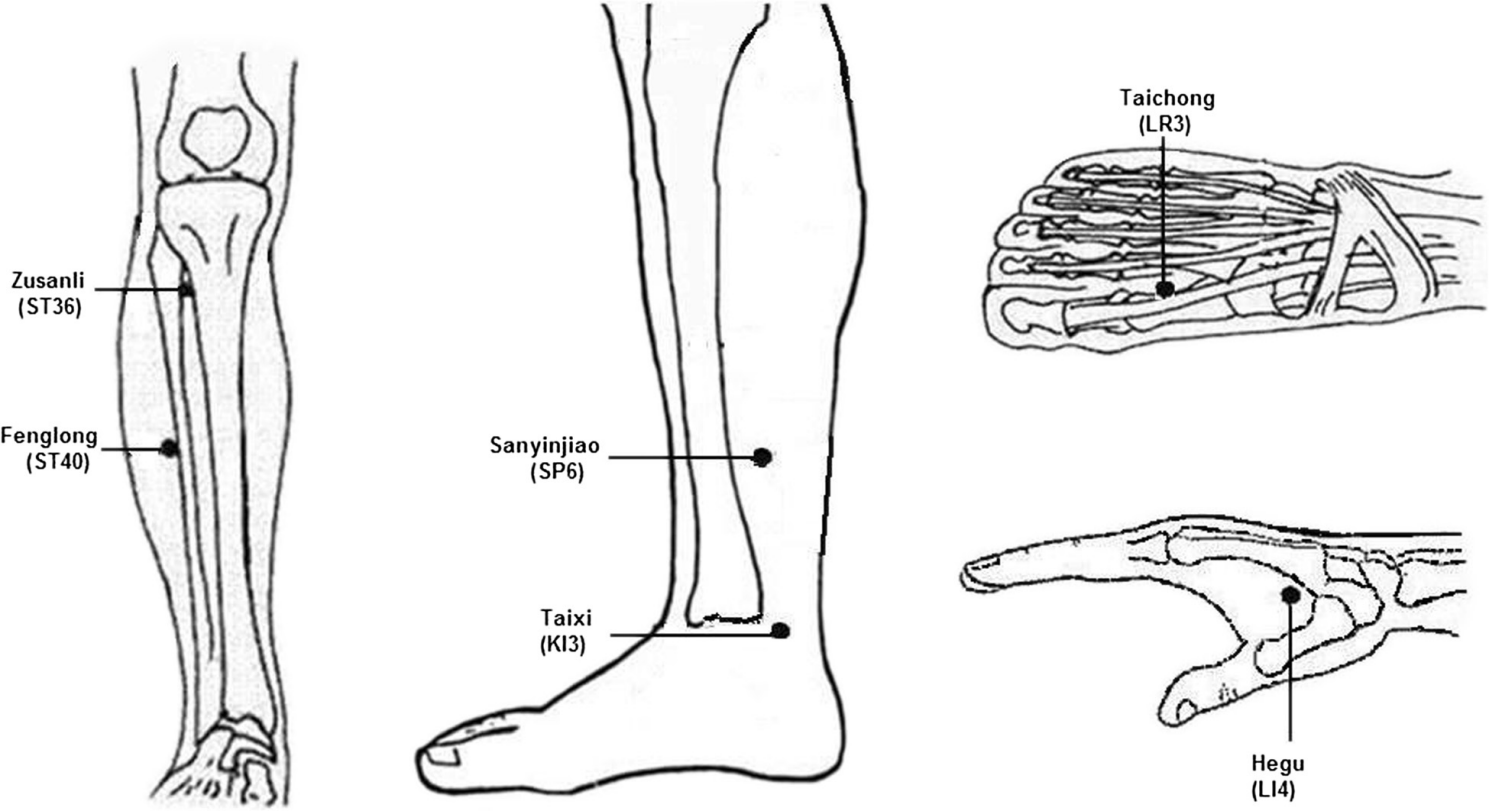
Acupoint locations.

All of the participants will undergo traditional Chinese medicine (TCM) diagnosis. TCM diagnosis in the great majority of infertile patients is kidney and spleen deficiency, liver *qi* stagnation, dampness, or blood stasis, or multiple diagnoses. Therefore, acupoints for the TEAS and EA groups are chosen according to the TCM dialectical treatment principle and our former experimental results [30]. The main acupoints are bilateral Zusanli (ST36), Sanyinjiao (SP6) and Taichong (LR3) (Figure 1). ST36 is an important health care acupoint of the stomach meridian, which can enhance spleen and stomach function and help generate *qi* and blood. SP6 is the confluent acupoint of foot three (kidney, spleen and liver) *yin* meridians, which can supplement the three *yin*s and can regulate *qi* and blood. LR3 is the liver meridian *yuan*-source acupoint, which can assist SP6 to soothe the liver and regulate *qi* and blood. Bilateral Taixi (KI3) will be added for patients with kidney deficiency, Hegu (LI4) will be added to assist Taichong (LR3) for patients with liver *qi* stagnation, and Fenglong (ST40) is for patients who have too much dampness in the body. Therefore, eight acupoints will be used for treating each patient. The combination of ST36, SP6 and LR3 can guarantee basic regulation of *qi* and blood throughout the body. Subsequent modification according to the syndrome differentiation meets the demand of the highlighted syndrome.

The TEAS or EA groups will receive a conventional IVF procedure with the addition of TEAS or EA at acupoints. For the EA group, sterile, disposable needles made in Shanghai (China) will be used to a depth of 5 to 20 mm in the selected acupoints after disinfection. The needles will be 25 to 50 mm in length and 0.3 mm in diameter. The *qi* sensation will be achieved by lifting and thrusting movements combined with needle twirling and rotation. Two homolateral acupuncture needles will be connected to form a circuit containing a HANS-100B stimulator (Nanjing Jisheng Company, China) for 30 min. The TEAS group will use skin electrodes placed on acupoints instead of piercing the skin with needles. The TEAS or EA parameters are as follows: alternating 2/100 Hz frequency wave, moderate 0.8 to 1.0 mA electrical current for EA, and 3 to 5 mA for TEAS with a 30×30 mm skin electrode.

TEAS or EA treatments will start from day 3 of menstruation in the ovarian stimulation cycle once every two or three days, 30 min each for more than two weeks until the day of ET. Women in the control group will receive only the conventional IVF procedure.

In total, two acupuncturists from each hospital with a minimum of two years of clinical experience will participate in this process. Training in the treatment protocol, study intent or treatment aspirations, treatment recording, and an explanation of proposed future monitoring will be provided to the acupuncturists.

### Outcome measures

The primary outcome measure is clinical pregnancy (confirmed by ultrasound scan 5 to 6 weeks after ET demonstrating at least one gestational sac with fetal heart activity).

Secondary outcome measures are live birth (defined as the delivery of one or more living infants, >20 weeks gestation or 400 g or more birth weight), number of oocytes retrieval (OCR) and the total gonadotropin dose used in the ovarian stimulation cycle. Ovarian responses are defined according to three categories: poor response, three or fewer OCR; average response, 4 to15 OCR; hyper-response, more than 15 OCR.

Clinical data will be collected from the IVF center databases and from the patients by following up at 6 and 40 weeks after ET. Data on TEAS or EA safety as well as any adverse events will be collected from the practitioner treatment notes.

### Quality control

To guarantee the study quality, all of the researchers will be required to attend all of the training classes and pass the training test. They must master all of the details of this trial before performing it. For instance, they must master using the randomization method and filling in the case report form (CRF). The acupuncturists should also master finding the correct points and manipulating the needles and electro-acupuncture apparatus. The assessors should master how to collect and input data accurately and completely.

During the trial, all of the details including all adverse events such as broken needles, bleeding, hematomas, fainting, serious pain, and local infection will be recorded. Serious adverse events will be immediately reported to the principal investigator, and rescue procedures will be initiated immediately.

To ensure trial quality, a clinical monitor designated by the principal investigator will verify all of the process details at regular intervals. Moreover, the monitor will check the authenticity of the data.

### Statistical analysis

Baseline subject characteristics are described by descriptive analysis, and the balance among groups or subgroups was assessed by analysis of variance or *χ*^2^-test. To assess effects of TEAS and EA, the *χ*^2^-test will be applied to compare frequencies between groups such as the CPR and LBR, and analysis of variance will be performed to compare continuous parameters (gonadotropin dose for example) between the groups (*α* = 0.05). All of the analyses will be based on both an intention-to-treat analysis (ITT, with all randomized patients) and a treated-per-protocol analysis (TPP, with all randomized patients minus withdrawals). Missing patient data for those that dropped out of the study will be analyzed using the last observation carried forward method. Patients with complete data missing for primary outcomes will be considered as a negative ITT pregnancy result and eliminated in the TPP analysis.

## Discussion

IVF is an option for many infertile women, their families and the public, but because of the relatively low success rate, high cost, and possible latent safety problems, new therapies that can improve pregnancy outcomes are highly desirable.

Acupuncture is a frequently used adjunctive therapy for many diseases. The application of acupuncture to IVF has attracted more doctors and patients. Based on the published data, this study will provide significant evidence for using a new method (TEAS) with IVF to improve reproductive outcomes. Although one trial [28] has already reported that TEAS significantly improved the clinical outcome of ET, stimulus frequency, treatment courses or times can be further optimized. Therefore, with appropriate intervention times (from the time of COH to the day of ET), enough treatment courses (six or seven sessions), syndrome differentiation and treatment according to individual characteristics, TEAS may produce a pleasantly surprising result.

In this protocol, we will select the intervention time from COH to ET, from day 3 of the ovarian stimulation cycle to the day of ET to obtain better results. Reasons for this are as follows: 1) the latest meta-analysis demonstrated that acupuncture at approximately the time of COH is the most suitable; 2) according to the studies of Paulus 2002 [4], Westergaard 2006 [7] and Zhang 2011 [28], acupuncture on the day of ET can significantly improve the reproductive outcome of IVF/ICSI; and 3) correcting the state of infertility caused by long-term insufficiency or imbalance requires enough treatment courses.

The control group was based on the fact that any surface stimulation at acupoints, such as acupressure, can cause a physiological reaction or effect [16]. Thus, the control group will not receive any sham TEAS or acupuncture, and they are included mainly as a control group for providing a baseline pregnancy rate. To avoid the psychological placebo effect, every participant will be informed that the TEAS or EA treatments are safe but that procedure effectiveness is unknown.

TEAS or EA frequency is an important issue for consideration. The alternating wave is the most frequently used wave in clinical acupuncture. Therefore, in this trial, we will determine an alternating wave with a relatively broad frequency range (2/100 Hz) for the TEAS or EA intervention. The electrical current is moderate at 0.8 to 1.0 mA for EA and 3 to 5 mA for TEAS based on patient comfort and will not reach the sustain threshold but can obviously be perceived by the patients.

## Trial status

This trial is currently preparing to recruit participants. The enrollment will begin on 1 May 2014 and is expected to be completed before 1 May 2016.

## Abbreviations

CAM: complementary and alternative medical; COH: controlled ovarian hyperstimulation; CPR: clinical pregnancy rate; CRF: case report form; EA: electro-acupuncture; ET: embryo transfer; FSH: follicle stimulating hormone; GnRH: gonadotropin-releasing hormone; hCG: human chorionic gonadotropin; ICSI: intracytoplasmic sperm injection; ITT: intention-to-treat analysis; IVF-ET: in vitro fertilization-embryo transfer; LBR: live birth rate; OA: oocyte aspiration; OCR: oocytes retrieval; PID: pelvic inflammatory disease; RCTs: randomized controlled trials; TCM: traditional Chinese medicine; TEAS: transcutaneous electrical acupoint stimulation; TPP: treated-per-protocol analysis.

## Competing interests

The authors declare that they have no competing interests.

## Authors’ contributions

CHZ, JZ, JW, and MMZ all contributed to developing the study protocol. CHZ drafted the manuscript, and all of the authors contributed to writing of the manuscript. All authors read and approved the final version.
